# Can You Hear Out the Melody? Testing Musical Scene Perception in Young Normal-Hearing and Older Hearing-Impaired Listeners

**DOI:** 10.1177/2331216520945826

**Published:** 2020-09-08

**Authors:** Kai Siedenburg, Saskia Röttges, Kirsten C. Wagener, Volker Hohmann

**Affiliations:** 1 Department of Medical Physics and Acoustics and Cluster of Excellence Hearing4all, Carl von Ossietzky University of Oldenburg; 2 Hörzentrum Oldenburg GmbH & Hörtech gGmbH, Oldenburg, Germany

**Keywords:** music perception, hearing impairment, auditory scene analysis, melody, pitch, timbre

## Abstract

It is well known that hearing loss compromises auditory scene analysis abilities, as is usually manifested in difficulties of understanding speech in noise. Remarkably little is known about auditory scene analysis of hearing-impaired (HI) listeners when it comes to musical sounds. Specifically, it is unclear to which extent HI listeners are able to hear out a melody or an instrument from a musical mixture. Here, we tested a group of younger normal-hearing (yNH) and older HI (oHI) listeners with moderate hearing loss in their ability to match short melodies and instruments presented as part of mixtures. Four-tone sequences were used in conjunction with a simple musical accompaniment that acted as a masker (cello/piano dyads or spectrally matched noise). In each trial, a signal-masker mixture was presented, followed by two different versions of the signal alone. Listeners indicated which signal version was part of the mixture. Signal versions differed either in terms of the sequential order of the pitch sequence or in terms of timbre (flute vs. trumpet). Signal-to-masker thresholds were measured by varying the signal presentation level in an adaptive two-down/one-up procedure. We observed that thresholds of oHI listeners were elevated by on average 10 dB compared with that of yNH listeners. In contrast to yNH listeners, oHI listeners did not show evidence of listening in dips of the masker. Musical training of participants was associated with a lowering of thresholds. These results may indicate detrimental effects of hearing loss on central aspects of musical scene perception.

Music listening typically means listening to sound mixtures. These mixtures are composed of sounds from multiple musical instruments or voices that superimpose in time and frequency. In a concert of orchestral music, for instance, one may find the stage populated by several dozens of musicians, exposing the audience to counterpunctual movements of melodies, layerings of various musical elements, dense textures, and combinations and contrasts of tone colors. Listeners must then infer a grouping structure from a musical scene, which in the simplest case could be melody and accompaniment, akin to a painting’s foreground and background. In what seems to be an effortless process, these musical elements are organized by the human auditory system according to principles of auditory scene analysis (ASA; Bregman, 1990). These principles yet may cause difficulties for individuals with hearing loss. If listening to, say, a violin concerto, a valid question is whether moderately hearing-impaired (HI) listeners are still able to hear out the solo violin in the midst of the rich orchestral accompaniment.

Research has long acknowledged the fundamental role of ASA in music listening (Bregman, 1990; McAdams & Bregman, 1979). ASA affects the experience of a whole gamut of musical attributes based on melody, harmony, timbre, and rhythm (Russo, 2019). More specifically, it has been shown that traditional voice leading rules of music composition implicitly improve the perceived independence of concurrent voices by virtue of ASA principles (Huron, 2001, 2016). ASA also is at the heart of orchestration techniques and determines the choice, combination, and arrangement of instruments to create a musical effect desired by musicians (McAdams, 2019). Auditory grouping of musical voices and melodies has further been described as a critical problem for listeners with cochlear implants (Limb & Roy, 2014; Paredes-Gallardo et al., 2018; Pons et al., 2016). However, research on music perception has not addressed the effects of moderate forms of hearing impairment on musical scene analysis. This is remarkable, given ASA’s critical role in hearing impairment: Disentangling simultaneous streams of sound—such as voices at a crowded (cocktail) party (Cherry, 1953)—is the key challenge for HI individuals. Anecdotal evidence suggests that musicians with hearing aids have problems in hearing (and coordinating with) fellow musicians in ensemble performance (Association of Adult Musicians with Hearing Loss, 2016). A survey study indicates that hearing aid users complain about a lack of musical sound quality, clarity, and distortions when listening to music (Madsen & Moore, 2014). However, the ways in which musical ASA is conducted by listeners with mild to moderate hearing loss—the vast majority of impairments—have not been studied in detail so far. Here, we present an experiment that taps into two central faculties of music listening: the perception of pitch sequences (or melodies) and the perception of timbre.

## Perceptual Underpinnings of Scene Analysis

A critical function of ASA is to group the sensory representations of sound sources that may overlap in time and frequency with other sound sources. Grouping criteria include frequency harmonicity, spatial separation, and coherent modulation in amplitude or frequency (Bregman, 1990; Darwin, 1997). Sensorineural hearing impairment then worsens ASA not primarily because of lowered pure-tone sensitivity—as characterized by the audiogram—but because the sound representations of HI listeners are degraded in comparison with the representations of normal-hearing (NH) listeners. Degradations include poor frequency resolution (broader auditory filters), reduced dynamic range compression, reduced sensitivity to temporal fine structure, and impaired binaural auditory processing (Moore, 2007), which in turn impair the acuity of bottom-up processing by corrupting basic auditory grouping criteria. Examples include that HI listeners with poor fundamental frequency (*f*_0_) discrimination show smaller benefits from f_0_ differences compared with NH listeners in simultaneous vowel identification (Summers & Leek, 1998).

 When two vowels share the same *f*_0_, HI listeners perceive only the presence of one vowel, contrary to NH subjects who tend to hear two (Arehart et al., 2005), even though other research did not find a reduced effect of the ability to use differences in *f*_0_ or vocal tract cues in a sequential stream segregation task with speech sounds in HI compared with NH listeners (David et al., 2018). Research has further shown that NH listeners benefit from comodulations in a masking stimulus in detecting a tone (Verhey et al., 2003), whereas HI listeners do not (Ernst et al., 2010).

A central, yet intricate question in the study of hearing loss and ASA concerns the extent to which deficits are due to hair cell dysfunction, that is, cochlear hearing loss, or age-related decline of neural processing along the auditory pathway. Aging has been associated with suprathreshold auditory processing deficits independently of sensorineural hearing impairment (e.g., Eipert et al., 2019; Moore, 2015). Nonetheless, both factors impede suprathreshold auditory processing abilities, whether measured in psychoacoustical tasks (Kortlang et al., 2016) or with speech reception thresholds (SRTs; Goossens et al., 2017). In the present study, we tested younger NH (yNH) listeners and older HI (oHI) listeners, hence not attempting to disentangle the components of age and hearing loss, but rather to obtain a first estimate of the strength of the integrated effect.

Considering cognitive processes involved in ASA, it has been established that to prioritize and track sound sources over time, ASA is strongly affected by selective attention (Alain & Arnott, 2000; Shinn-Cunningham & Best, 2008; Woods & McDermott, 2015) and memory (Bey & McAdams, 2002; Woods & McDermott, 2018). Selective attention appears to be particularly accurate in musicians, as indicated by stronger event-related potentials in electroencephalography recordings from the human scalp during active listening tasks and better behavioral performance compared with nonmusicians (Zendel & Alain, 2013, 2014). Musicians outperform nonmusicians in an attentive tracking experiment (Madsen et al., 2019), and musicians appear to be better aware of ambiguity in ASA (Pelofi et al., 2017). Studies have even observed musical training to positively affect the ability to understand speech in noise (Dubinsky et al., 2019; Parbery-Clark et al., 2009; Puschmann et al., 2018; Slater et al., 2015; Zendel et al., 2019), although there is debate regarding the robustness of the effect (Boebinger et al., 2015; Madsen et al., 2019; Ruggles et al., 2014). Seeking to obtain a first estimate of NH and HI listeners’ performance in musical scene analysis tasks, we here used a task that required listeners to focus their attention on a target instrument playing a short tone sequence and to separate the contributions of the target and masker signals even for low target levels.

## Music Perception and Hearing Impairment

When it comes to how HI listeners perceive music, relatively little work has addressed moderate forms of hearing impairment in specific terms. Emiroglu and Kollmeier (2008) measured the discrimination of artificially morphed musical instrument sounds in quiet and with various types of stationary noise and observed that only HI participants with steeply sloping hearing loss showed worsened timbre discrimination abilities. However, it remained unclear whether these results generalized to realistic musical scenarios with much more complex types of musical sounds. More recently, Kirchberger and Russo (2015) provided a test battery to map out music perception of listeners with hearing impairment, encompassing subtests on the musical parameters of meter, harmony, melody, intonation, and timbre. Stimuli consisted of digitally synthesized sounds and were presented in isolation in most of the subtests, hence not accounting for musical ASA. As an exception, the so-called *melody-to-chord ratio* subtests provided a measure of musical ASA, as these subtests required listeners to match transposed four-note melodies that were presented with a chordal accompaniment. The battery was evaluated with NH listeners and with HI listeners with mild to moderate hearing impairments. The authors observed elevated discrimination thresholds of HI listeners in the seven subtasks that relied on forms of *f*_0_ and spectral envelope processing. Requiring listeners to match transposed melodies, the melody-to-chord ratio subtest appeared to be particularly difficult such that roughly a quarter of NH participants and a third of HI participants were not able to complete the task. Overall, the results from Kirchberger and Russo (2015) suggest that hearing impairment negatively affects the perception of isolated musical parameters distinguished by periodicity or spectral envelope information but that parameters based on amplitude level or temporal features could be unaffected.

Choi et al. (2018) observed that thresholds for the detection of joint spectrotemporal modulations measured for NH, HI, and cochlear implant listeners predicted accuracy in a pitch and melody discrimination task and even more precisely for instrument identification. However, the level of participants’ musical expertise was not controlled, which may explain some of the interindividual differences within the groups of hearing aid and cochlear implant users, and again these experiments did not touch on the role of ASA. Madsen et al. (2015) considered ratings of sound clarity from HI listeners, based on polyphonic musical excerpts that were processed with wide dynamic range compression, either applied to the mixture or to individual instruments only. They observed lower clarity with compression compared with linear amplification and no overall effect of compression speed. Although this result may help to improve strategies for hearing device fitting for music, it does not allow to assess the extent to which HI listeners’ perception of clarity of a musical scene is objectively different from NH listeners. Overall, this review suggests that HI listeners’ ASA abilities in realistic musical scenarios deserve further attention.

The main goal of the present study was to obtain a first estimate of the effect of moderate hearing impairment on musical scene analysis. We hence devised two scene analysis tasks that use a simple musical setting based on a diatonic melody-accompaniment scheme with recorded sound samples. Specifically, stimuli consisted of short four-note tone sequences played by a clarinet, flute, or trumpet (the target signal) that were to be identified in the presence of an accompaniment that was a dyad played by a piano or cello or a spectrally matched noise (the masker). We measured speech-in-noise reception thresholds as a control variable to discern whether thresholds from music and speech tasks would be associated. Based on the plethora of previous reports of reduced (nonmusical) ASA in oHI listeners, we expected to observe higher thresholds for pitch sequence and timbre matching of oHI listeners compared with yNH listeners. We also expected advantages for musically trained listeners. No specific hypotheses were formulated regarding differences across tasks and masker conditions.

## Participants

### Method

**Figure 1.**
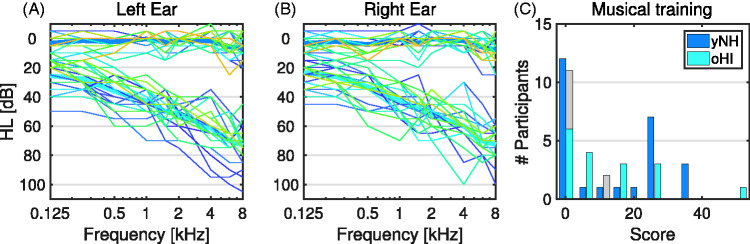
Participant specifications. Mean pure tone audiometric thresholds of younger normal-hearing (yNH, dark blue) and older hearing-impaired participants (oHI, light blue) are given in panels A (left ear) and B (right ear). Individual data is shown in thin colored lines. (C) Distribution of musical training scores as described in the text. Note that seven oHI participants (in gray) were removed from the analysis due to insufficient performance (see Appendix C).

This study recruited 28 yNH and 24 oHI participants who received monetary compensation for their time. One participant from the yNH group was discarded from the sample because of a pure-tone average (PTA) higher than 20 dB hearing level (HL); another yNH participant was discarded because it turned out this participant was not a German native speaker, which was problematic for the German speech intelligibility test. The remaining 26 yNH participants had a mean age of 26 years (*SD* = 6.9, range = 21−56 years) and a mean PTA (averaged across 0.5, 1, 2, and 4 kHz) of 1.6 dB HL (*SD* = 2.4, range = –2 − 10 dB HL). Figure 1 (Panels A and B) show the complete audiograms of all participants. The 24 oHI participants had a mean age of 69 years (*SD* = 3.9, range = 59 − 74 years) and a mean PTA of 47.6 dB HL (*SD* = 5.3, range = 38 − 58 dB HL). One oHI participant did not complete the retest session (but was included in the linear mixed-effects model).

Due to the substantial differences in age between the groups of yNH and oHI participants, musical experience of participants needed to be assessed in a way that was relatively independent of age—hence, single-item measures such as *number of years of instruction on an instrument* (cf. Zhang & Schubert, 2019) would risk to inaccurately portray many older participants as highly skilled musicians. We measured musical training using the corresponding self-report inventory of the Goldsmiths Musical Sophistication Index (Müllensiefen et al., 2014) but discarded an item that was particularly affected by age, namely the number of years of regular practice on an instrument (including the voice). The six remaining items were weighted as in the original index (see Appendix A for the complete list of items and weightings). Figure 1(C) shows the distribution of the resulting musical training scores for both groups of participants: There were 12 yNH and 11 oHI participants without any musical training according to this metric, as well as 14 yNH and 13 oHI participants with musical training. The median musical training score of yNH participants was 7.0 compared with 5.1 for oHI participants, but a Wilcoxon rank sum test did not indicate substantial differences between the two medians, *z* = 0.9, *p* = .36.

### Stimuli

**Figure 2.**
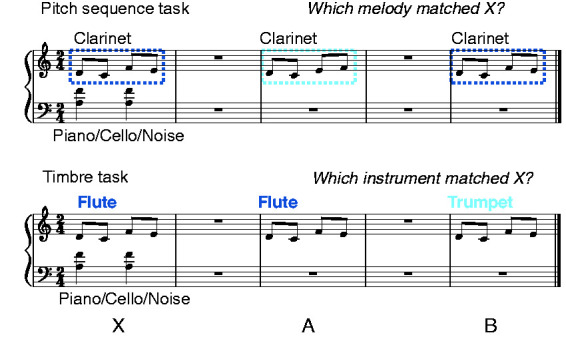
Example of stimuli in the two experimental tasks.

The main experiment comprised a pitch sequence and a timbre task. In both tasks, participants were presented with a mixture consisting of a target signal plus a masker, X, followed by two different versions of the target signal, A and B. The target signal in X either equalled A or B (half of the targets were A, and half were B). In the pitch sequence task, Signals A and B differed in terms of the sequential ordering of pitches, implemented through a swap of two tones, and all tones were clarinet sounds. In the timbre task, sounds in Signals A and B came from different instruments (transverse flute vs. trumpet), but both signals used the same pitch sequence. The maskers were dyads with sounds from the piano or the cello, or spectrally matched noise (see later). Figure 2 illustrates the two tasks in musical notation.

The temporal stimulus properties were structured as follows: the interstimulus interval, separating X, A, and B, had a length of 1 s. Maskers consisted of two dyads with 500-ms interonset interval (corresponding to quarter notes at a tempo of 120 beats per minutes). Melodies consisted of isochronous four-tone sequences at twice the rate of the maskers, that is, with 250-ms interonset intervals (corresponding to 8th notes).

Regarding the presented musical pitch structures, stimuli were built around a central pitch class that was drawn from the range of D4-F#4 (*f*_0_: 294–370 Hz). Any such center pitch class was part of six triad chord types (major/minor in three inversions); the masker chord consisted of the two outer pitch classes of these triads. Tone sequences were built from four distinct pitch classes that included the center pitch class in conjunction with three other pitch classes from diatonic scales (corresponding to the major/minor chord). Pitch classes from the sequence could match the chord pitch classes but did not exceed the range of the chord pitch classes (at min. A3, *f*_0_: 220 Hz, max. B4, *f*_0_: 494 Hz). This means, the target and the masker did not excite separable critical bands. The sequential order of these tones was fully randomized. In the pitch sequence task, Sequences A and B differed in terms of a swap of the order of sounds at Positions 2 and 3 or 3 and 4, but the swaps that were used always led to exactly one violation of contour between Melodies A and B.

**Figure 3.**
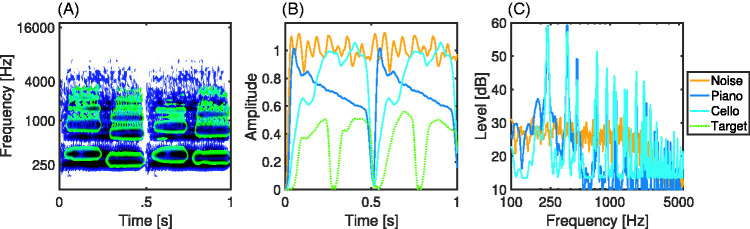
Visualization of acoustic properties of the stimuli. (A) Illustration of morphology of signals and maskers: Auditory spectrogram with flute target sounds (green) plotted on top of the cello masker sounds (blue). (B) Amplitude envelopes of exemplary masker sounds. The dotted green line shows corresponds to a target signal (here clarinet) at -6 dB signal to masker ratio. (C) Smoothed frequency spectrum of masker sounds.

Sound samples were recordings of isolated tones played on acoustic musical instruments from the Vienna Symphonic Library.^1^ Only the left channels were used from the stereo samples. All sounds were low-pass filtered using a finite impulse response filter with cutoff frequency of 8 kHz and a band-stop frequency of 10 kHz with 65 dB attenuation. The individual tones from the flute, trumpet, and clarinet were played at forte corresponding to a duration of 250 ms. The masker tones from the cello and piano were played at forte dynamics and conceived as 8th-notes at a tempo of 120 quarter notes per minute, dynamics as quarter notes, yielding sounds with a duration of 500 ms. As additional noise masker, stationary noise was used that was matched in terms of its smoothed long-term spectral envelope (root-mean-squared average) with the test sounds from the three target instruments (flute, trumpet, and clarinet). This stimulus was also used for the loudness scaling task. A visualization of the amplitude envelope and frequency spectra of exemplary maskers is shown in Figure 3(B and C).

For measuring speech intelligibility, the Oldenburg sentence test was used (*Oldenburger Satztest*; Wagener & Brand, 2005; Wagener et al., 1999a, 1999b, 1999c), which has a battery of prerecorded and fine-tuned matrix sentences in German language.

### Procedures

The procedure was approved by the ethics committee of the University of Oldenburg. The experiment was administered in two sessions on separate days. The first session comprised the following subtasks: (a) loudness scaling, (b) timbre matching, (c) pitch sequence matching, (d) Oldenburg sentence test, and (e) a questionnaire on biographic information and musical training, and the second session comprised the following subtasks: (a) timbre matching and (b) pitch sequence matching. The order of these tasks was kept fixed for all participants.

The order of the pitch sequence and timbre matching tasks was not randomized because we noticed in pilot experiments that switching from the pitch sequence to the timbre task appeared to be very demanding, but not the other way around, potentially because participants are used to comparing musical pitch sequences (that is, melodies), but not so musical timbres. Randomizing the order of these tasks may thus have severely distorted the reliability of the measurement of the timbre task for half of the participants, which we sought to avoid. In the following, the procedures applied in the specific subtasks are described in greater detail.

#### Loudness Scaling

To be able to individually adjust the loudness of stimuli for oHI participants, a loudness scaling experiment was conducted according to the Adaptive Categorical Loudness Scaling procedure (Brand & Hohmann, 2002). In every trial, participants rated the perceived loudness of a spectrally matched noise on a scale from *inaudible* to *too loud*, and the subsequent presentation levels were selected adaptively with an upper limit of 90 dB sound pressure level (SPL). This upper limit was smaller compared with the original work (115 dB in Brand & Hohmann, 2002) and was chosen because we wished only to estimate the medium loudness, not the whole loudness function. The resulting medium loudness level, corresponding to 25 CU, was estimated by using the BTUX fitting method (Oetting et al., 2014).

#### Pitch Sequence and Timbre Matching

The signal level was varied in a two-down/one-up staircase procedure that converges to a signal-masker level ratio with 71% correct responses (Levitt, 1971). The initial step size was 8 dB which was halved after every second reversal with a minimum step size of 2 dB. Tracks were terminated after 12 reversals, and the threshold was defined as the arithmetic mean of the last 8 reversals.

The center pitch class and the chord type were both roving variables that were selected randomly throughout tracks. The masker type was changed blockwise, that is, it stayed fixed within each track. Both the timbre and the pitch sequence matching task were preceded by explanations of the task through the experimenter and by six training trials that could be repeated, if participants wished to do so.

#### Speech Intelligibility

Measurements of speech intelligibility in noise followed the standard protocol of the Oldenburg sentence test (Wagener & Brand, 2005; Wagener et al., 1999a, 1999b, 1999c). In brief, participants were presented with one five-word sentence per trial and were instructed to report every intelligible word to the experimenter. Concurrent to the sentences, stationary speech-shaped masking noise was presented at a fixed level of 65 dB SPL. An adaptive procedure adjusted the speech signal level to approach the 50% threshold of speech intelligibility (Brand & Kollmeier, 2002). We measured two lists of sentences with 20 sentences each. The first list was treated as training and the second list as the measurement.

### Presentation and Apparatus

For the pitch sequence and timbre matching tasks, the presentation level of the masker was held fixed at 65 dB SPL for yNH participants and at medium loudness (25 CU) for oHI participants, rounded in 5 dB steps, but not more than 80 dB SPL. The maximal possible signal level was limited to 90 dB SPL; for participants with maximal masker level (80 dB), the maximal signal-to-masker (SMR) ratio thus was 10 dB. The main experiment comprised 13 oHI participants with a masker level of 80 dB, 4 oHI participants with 75 dB, 4 oHI participants with 70 dB, and 3 oHI participants with 65 dB SPL. See Appendix B for further information and discussion of the role of the masker level in the present study.

Participants were tested individually in a sound-proof lab and provided responses on a computer keyboard. Sounds were presented through The Mathworks MATLAB and were DA converted with an RME Fireface audio interface at an audio sampling frequency of 44.1 kHz and 24-bit resolution. Sounds were presented diotically over Sennheiser HDA 650 headphones. The masker level was calibrated by a Norsonic Nor140 sound-level meter with a G.R.A.S. IEC 60711 artificial ear to which the headphones were coupled.

### Data Analysis

Empirical thresholds were analyzed using linear mixed models (West et al., 2007). All mixed-effects analyses were conducted with the software *R* (3.5) using the packages *lme4* (Bates et al., 2014). As recommended by Barr et al. (2013), our model included a full crossed random effects structure for participants and test session, that is, by-participant intercepts and slopes for the task and masker variables and their interaction, as well as by-session intercepts and slopes for the group and training variable. All categorical predictors were sum-coded. For the masker factor, this meant that both the piano and the cello maskers were contrasted with the noise masker. The musical training score was used as a continuous predictor. The data can be made available upon request. The key analysis results are provided as part of Table D1 in Appendix D. The table includes *p* values adjusted for multiple comparison within the linear model (Cramer et al., 2016), using the false discovery rate (Benjamini & Hochberg, 1995). Marginal means and confidence intervals (CIs) as provided in the text were estimated from the fitted models using the *emmeans* package (Lenth, 2018). Concerning the statistical evaluation, we follow the current recommendation from the *American Statistical Association* (Wasserstein et al., 2019) by refraining from dichotomizing statistical significance based on thresholded probability values (*p* < .05) and rather describe the empirical results in quantitative terms.

**Table D1.** Linear Mixed Model Estimates With Full Crossed Random Effects: Fixed Effects (Marginal) *R*^2^ = .57; Fixed and Random Effects (Conditional) *R*^2^ = .77.

	β	CI		*t*	*p*	*p* adj.
		Low	High			
Intercept	–10.5	–12.5	–8.4	–10.4	.016	.029
Group	–5.0	–6.0	–3.9	–9.2	<.001	<.001
Task	2.0	1.2	2.7	5.0	<.001	<.001
Masker (Piano)	–3.8	–4.5	–3.1	–10.7	<.001	<.001
Masker (Cello)	2.3	1.7	2.9	7.9	<.001	<.001
Training	–1.6	–2.7	–0.4	–2.9	.011	.024
Group:Task	–1.0	–1.9	–0.2	–2.6	.013	.026
Group:Masker (Piano)	–3.5	–4.2	–2.9	–10.0	<.001	<.001
Group:Masker (Cello)	1.2	0.6	1.7	4.0	<.001	<.001
Task:Masker (Piano)	–1.5	–2.0	–0.8	–4.7	<.001	.024
Task:Masker (Cello)	0.8	0.2	1.4	2.7	.010	<.001
Group:Training	–0.4	–1.5	0.7	–0.7	.488	.558
Task:Training	0.3	–0.5	1.1	0.7	.476	.558
Masker (Piano): Training	–0.6	–1.3	0.1	–1.7	.093	.539
Masker (Cello): Training	0.2	–0.3	0.8	0.8	.404	.160
Group:Task:Masker (Piano)	–1.5	–2.1	–0.9	–4.8	<.001	.009
Group:Task:Masker (Cello)	1.0	0.3	1.5	3.1	.003	<.001
Group:Task:Training	–0.4	–1.1	0.4	–1.0	.336	.482
Group:Masker (Piano):Train	–0.1	–0.8	0.6	–0.3	.776	.482
Group:Masker (Cello):Train	–0.3	–0.9	0.3	–1.0	.330	.810
Task:Masker (Cello):Train	0.3	–0.3	0.9	1.0	.342	.482
Task:Masker (Piano):Train	0.1	–0.5	0.8	0.4	.709	.774

*Note*. The rightmost column lists *p* values adjusted for multiple comparisons (false discovery rate method). CI = confidence interval.

## Results

Among the 24 oHI participants, 7 participants achieved levels of performance that were not sufficient to reliably measure 71%-correct SMR thresholds. Note that among these seven participants, five participants did not have had any musical training. For that reason, these participants were excluded from the data visualization and analysis (see Appendix C for details).

**Figure 4.**
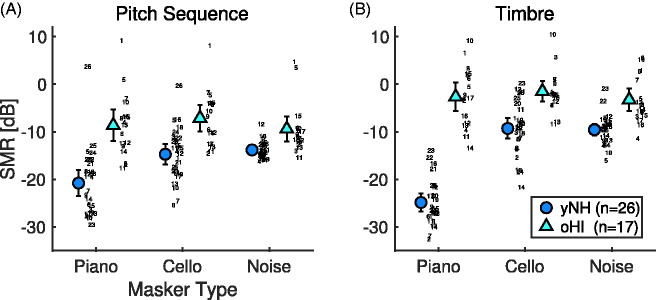
Distributions of thresholds for pitch sequence task (A) and timbre task (B). The legend indexes the groups of younger normal-hearing (yNH) and older hearing-impaired (oHI) participants. Individual data is plotted as subject ID (per group). Errorbars correspond to 95% confidence intervals.

Figure 4 depicts the distribution of SMR thresholds for the pitch sequence and timbre tasks for all experimental conditions, averaged across test and retest session. Correlating the average thresholds per participant across sessions yielded a test–retest correlation of *r* = .72 (CI [0.62, 0.79]) for the pitch sequence task and *r* = .74 (CI [0.65, 0.81]) for the timbre task. In the pitch sequence task, mean thresholds ranged between around –20 and –14 dB SMR for yNH participants and between –9 and –7 dB for oHI participants. In the timbre task, mean thresholds ranged between –25 and –9 dB SMR for yNH and –4 and –2 dB for oHI participants.

The statistical analysis indicated strong main effects for the factors of group (yNH, oHI), task (pitch sequence, timbre), and masker (piano, cello, noise; all |β| > 2.0, see Table D1). Estimated marginal means indicated differences of around 10 dB in thresholds without overlap of 95% CIs between yNH (*M* = –15.4 dB, CI [–18.9, –11.9]) and oHI participants (*M* = –5.5 dB, CI [–10.2, –0.8]).

**Figure 5.**
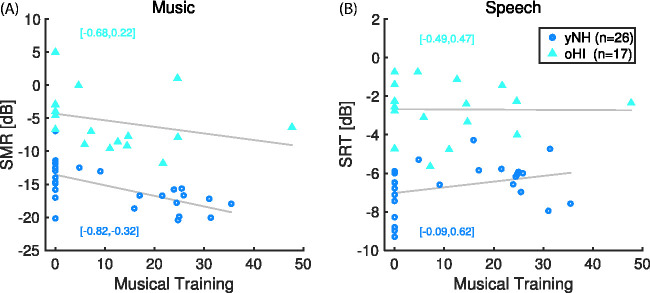
Association of musical training index with (A) signal-to-masker (SMR) thresholds averaged across pitch sequence and timbre task, and (B) speech reception thresholds (SRT). 95% confidence intervals of correlation coefficients are given in brackets.

Musical training was associated with a lowering of thresholds (β = –1.6, CI [–2.7, –0.4]), that is, every unit in the *z*-normalized musical training scores led to a lowering of 1.6 dB in thresholds. If considered on a group level by splitting participants with training scores above zero from the rest, this implied lower thresholds of participants with musical training (*M* =–12.0 dB, CI [–14.9, –9.1]) compared with participants without any musical training (*M* = –8.7 dB, CI [–14.3, –2.0]). Figure 5 shows the correlations of musical training scores separately for the groups of yNH and oHI participants for the pitch sequence and timbre tasks averaged across masker and tasks conditions (Panel A) as well as for the speech intelligibility task (Panel B). The correlation of the musical training index and the averaged thresholds from the music tasks amounted to *r* = –.63, CI [–0.82, –0.32] for yNH participants and *r* = –.29, CI [–0.68, 0.22] for oHI participants. Thus, the association of training and a decrease of thresholds was particularly pronounced for yNH participants (but note that the analysis using the linear mixed model did not indicate any strong interactions between musical training and task or group).

A small difference of thresholds arose across tasks, where the pitch sequence task (*M* = –12.4 dB, CI [–16.3, –8.5]) yielded thresholds around 3 dB lower compared with the timbre task (*M* = –8.5 dB, CI [–13.1, –3.9]) with large overlap of CIs. Finally, thresholds for the piano masker (*M* = –14.2 dB, CI [–18.0, –10.5]) were lower compared with both the cello masker (*M* = –7.3 dB, CI [–11.6, –2.9]) and the noise masker (*M* = –8.1 dB, CI [–12.5, –3.8]), but the difference between cello and noise condition seems to be of minor importance, given less than 1 dB of a difference and mostly overlapping CIs.

Importantly, the analysis suggested interactions with rather strong effect sizes (|β| > 1) between the factors of masker and group, as well as masker and task. These interactions appear to be driven by the three-way interaction between group, task, and masker (piano; β = –1.5, CI [–2.1, –0.9]). This interaction may be considered from the following perspective: yNH participants had higher thresholds for the pitch sequence task with the piano masker (*M* = –20.7 dB, CI [–24.1, –17.4]) compared with the timbre task with the piano masker (*M* =–24.8 dB, CI [–27.8, –21.7]), paired *t*(25) = 2.8, *p* = .009 (here and in the following, *p* values were Bonferroni–Holm corrected for multiple comparisons, *n* = 6), but there were lower thresholds in the pitch sequence task with cello (*M* = –14.7 dB, CI [–17.7, –11.6]) and noise maskers (*M* = –13.8 dB, CI [–16.9, –10.6]) compared with the timbre task with cello (*M* = –9.2 dB, CI [–12.3, –6.2]) and noise maskers (*M* = –9.5 dB, CI [–12.7, –6.3]), *t*(26) > 3.9, *p* < .003. On the contrary, there was no reversal of the effect for oHI participants, who had consistently lower thresholds for the pitch sequence task compared with the timbre task, *t*(16) >3.4, *p* < .004. This means, yNH participants were exceptionally good in matching timbre in the presence of the impulsive piano masker, but for oHI participants, the masker type did not make a substantial difference.

SRTs were 4 dB lower for yNH participants (*M* = –6.7 dB signal-to-noise ratio [SNR], CI [–6.8, –6.5]) compared with oHI participants (*M* = –2.7 dB SNR, CI [–2.9, –2.5]), accompanied by a robust separation of CIs. As visible in Figure 5(B), however, there was no linear correlation between musical training scores and SRTs for yNH or oHI participants. That means, musical training was generally associated with a lowering of pitch sequence and timbre thresholds but not of speech intelligibility thresholds.

**Figure 6.**
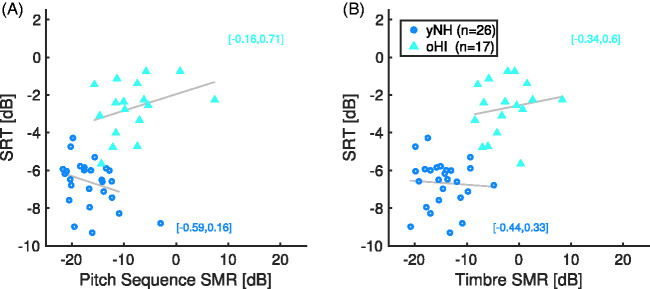
(A) Relation between SRT and signal to masker ratios (SMR) for pitch sequence tasks averaged across the three maskers. The regression excluded one yNH participant (#27) with average thresholds near 0 dB. (B) Relation between SRT and SMR in the timbre task. Regression lines were computed separately for the groups of yNH and oHI participants. 95% confidence intervals of correlation coefficients are given in brackets.

Finally, to consider associations of speech recognition and musical scene analysis thresholds, we correlated the thresholds across tasks. We computed correlations separately for the group of yNH and oHI participants to account for the potential common confounder of hearing impairment. Figure 6 provides the corresponding scatterplot between the speech recognition scores and the thresholds of the pitch sequence and timbre task, averaged across the three different maskers. The plot also contains linear regression estimates (gray lines) for the two groups of yNH and oHI participants. Notably, there was no correlation that was robustly different from zero. There was a tendency for pitch sequence SMR and SRT to show a negative association for yNH participants (*r* = –.40, *p* = .040, CI [–0.69, –0.02]), but after removing an outlier with a pitch sequence SMR close to zero and an SRT of around –9 dB SNR, the correlation vanished (accordingly, the regression line in Figure 6 depicts the regression line after outlier removal). Considering this lack of an association between music and speech tasks, one could also argue that only the music tasks with the stationary noise masker would correspond to the stationary noise masker in the speech intelligibility task. However, neither the SRTs of yNH nor oHI participants correlated with thresholds in the pitch sequence or timbre task with the noise masker (*p* > .14). Hence, the present data do not suggest any notable associations of speech recognition scores and musical scene analysis abilities independent of participants’ basic hearing thresholds.

To summarize the main results, oHI participants yielded drastically higher pitch sequence and timbre SMR thresholds and more variability compared with yNH participants. Musical training was associated with a lowering of SMR thresholds in the pitch sequence and timbre tasks, but not of SRTs in the speech reception task. An interaction between the factors group, masker, and task indicated that yNH participants achieved particularly low thresholds for the piano masker in the timbre task, but oHI participants did not show any consistent differences across maskers.

## Discussion

ASA has traditionally been an important topic in music perception (Huron, 2001; McAdams & Bregman, 1979), but only little is known about musical scene analysis of HI listeners. Our results indicate around 10 dB differences in mean thresholds between yNH and oHI listeners, demonstrating striking differences in musical scene analysis abilities. This implies that oHI listeners can have severe problems with the ecologically relevant music perception tasks of hearing out a pitch sequence or identifying an instrument from a mixture. This quantitative result complements informal evidence from oHI musicians who have reported a lack of sound clarity and problems with playing in larger musical ensembles (Association of Adult Musicians with Hearing Loss, 2016). Specifically, the lack of level headroom led to the exclusion of seven HI listeners (most of them without musical training). It is to be noted that with sufficient headroom, these listeners would likely have yielded thresholds higher than the respective group average. Therefore, we interpret the present result as a rather conservative estimate of the difference between yNH and oHI listeners.

We acknowledge that the present effect of hearing impairment is confounded by the factor of age, which is well known to negatively affect suprathreshold auditory processing in its own right (e.g., Moore, 2015). Other studies showed that performance of older NH listeners is worse than that of yNH listeners but better than that of oHI listeners, as for example in the case of basic psychoacoustic tasks such as tone-in-noise detection and frequency modulation detection (Kortlang et al., 2016) as well as pitch and timbre processing (Bianchi et al., 2019; Kirchberger & Russo, 2015). With regard to speech perception, Goossens et al. (2017) observed differences in SRTs of 2, 4, and 9 dB between yNH listeners (mean age: 23 years) and older NH listeners (74 years) for stationary white noise, stationary white noise with 4 Hz amplitude modulation, and the international speech test signal, respectively. The latter was used because it induces strong informational masking comparable with the presence of a simultaneous speaker (Holube et al., 2010). Differences between yNH and younger HI listeners were greater with 6, 10, and 13 dB, and the integrated differences between oHI and yNH listeners amounted to 10, 14, and 20 dB for the three noise types, respectively. These results were interpreted as evidence for a particularly detrimental effect of informational masking for older listeners, but a smaller effect of age on speech reception in scenarios dominated by energetic masking. With regard to music perception, Bones and Plack (2015) indicated that older listeners rated consonant chords as less pleasant and dissonant chords as more pleasant compared with younger listeners. Using a neural consonance index derived from the electrophysiological frequency-following response, older listeners also had less distinct neural representations of consonant and dissonant chords. However, to the best of our knowledge, no attempts have yet been made to disentangle deficits related to cochlear hearing loss and age-related deficits of neural processing in music perception. The present thresholds may hence be interpreted as a first estimate of the upper and lower bounds of scene analysis abilities and constitute the first indication of severely reduced musical scene analysis in listeners with moderate hearing loss.

An aspect that deserves further discussion concerns the overall presentation level. Properly adjusting presentation levels for HI listeners can be difficult due to their drastically restricted dynamic range. Our rationale to ensure dynamic range for HI listeners was to increase the masker level to their individual perceived medium loudness level. We kept the level of the masker fixed at 65 dB SPL for NH listeners, for whom level was not assumed to play an important role. Note that there have been reports of increased pitch discrimination thresholds as a function of increasing presentation level (Bernstein & Oxenham, 2006). It could hence be argued that the generally lower presentation levels were beneficial for NH listeners. However, a control experiment presented in Appendix B justified our assumption that the presentation level did not seem to have any strong or consistent effect on thresholds of NH listeners.

Considering the two experimental tasks, listeners showed lower thresholds in the pitch sequence task compared with the timbre task with the exception of the piano masker. Although we cannot strictly rule out the role of order effects in this result (the timbre task preceded the pitch sequence task), we interpret this effect as likely due to listeners’ greater familiarity with matching melodies compared with timbres. The former task is in fact deeply ingrained in Western musical culture wherein every child learns to memorize short musical melodies. It has further been indicated that if pitted against each other, listeners instructed to attend to timbre are easily distracted by concurrent melodic variation (Krumhansl & Iverson, 1992; Siedenburg & McAdams, 2017, 2018). Moreover, the perceptual salience of reordering pitch sequences could be greater compared with the salience of the timbral differences between the trumpet and the flute (Moore & Gockel, 2012). In any case, it should be noted that the differences between the pitch sequence and timbre tasks are rather small in comparison with other effects observed in the experiment, and both tasks elicited a similar range of thresholds and hence both have proven to be suited to study musical scene analysis. Future work could extend the current paradigm by using other musically more complex masker stimuli and account for the role of spatial separation between target signal and masker as well as potential room acoustical effects.

In addition to the general quantitative difference between yNH and oHI listeners, oHI listeners were qualitatively different from yNH participants in the sense that on average they were unable to improve their thresholds for the impulsive piano masker—which may be considered as an indication of dip listening by yNH listeners. In the general psychoacoustic literature, dip listening is a thoroughly documented phenomenon (Buus, 1985; Russo & Pichora-Fuller, 2008; Verhey et al., 2003), wherein a local increase in SNR is exploited by yNH listeners for detecting a signal in an amplitude-modulated masker. Notably, oHI listeners have been reported to show smaller release from masking for comodulated maskers (Ernst et al., 2010). As illustrated in Figure 3(A), in the present study, the maskers and signals substantially overlapped in time-frequency space. But the piano masker was decaying impulsively and hence exhibited much higher SMR ratios toward the end of the sound, as is shown in Figure 3(B). Our interpretation of these results is that yNH listeners were able to exploit this stimulus feature and achieved impressive thresholds of around –20 dB for the pitch sequence task and even –25 dB SMR for the timbre task (correspondingly, the statistical analysis yielded a strong three-way interaction between the factors of group, task, and masker). The timbre task may have even better allowed for successful listening in the dips because it only required to identify the right instrument only from one of the four sequence tones, whereas the pitch sequence task required listeners to extract the full pitch sequence from the mixture. It is possible that yNH listeners require only sparse information comparable to auditory glimpses (Cooke, 2006; Josupeit et al., 2018) to identify the instruments present in a mixture—a potential feature of the healthy auditory system that musicians may take for granted when building dense music compositions and productions.

Musically trained listeners had on average around 3 dB lower thresholds compared with listeners without explicit musical training, even though the matching tasks by themselves did not require any music theoretical or practical musicianship skills. This finding aligns with the literature on differences in auditory processing between musicians and nonmusicians (e.g., Herholz & Zatorre, 2012; Patel, 2008). These advantages even seem to extend for oHI listeners as Bianchi et al. (2019) recently demonstrated enhanced temporal fine structure and pitch processing in musically trained younger and older listeners with or without hearing impairment. The generality of the musician advantage remains contested, however. Although some authors have suggested superior auditory perception of musicians even in speech recognition tasks (Dubinsky et al., 2019; Parbery-Clark et al., 2009; Patel, 2014; Puschmann et al., 2018; Zendel et al., 2019), other studies were unable to replicate a consistent musician advantage in speech recognition (Madsen et al., 2017; Ruggles et al., 2014). Recently, Madsen et al. (2019) observed a musician advantage in purely auditory tasks such as pitch discrimination and interaural time discrimination, but no advantage was observed for speech recognition, suggesting that the musician advantage pertains to purely auditory tasks, but not to speech processing. It is to be noted that we did not observe an association between SRTs and musical training. More important, we did not observe a consistent correlation between musical scene analysis tasks (pitch sequence and timbre matching) and speech recognition, if hearing impairment was accounted for. Hence, we interpret our results as suggesting a musician advantage that only extends within the habitat of musical scene analysis, consistent with the auditory-specific musician advantages observed by Madsen et al. (2019).

## Conclusion

In this study, we compared the musical scene analysis abilities of yNH and oHI listeners using a pitch sequence and timbre task with three different masker types. oHI listeners with a moderate impairment had severe difficulties in *hearing out* melodies or instruments from a musical mixture as indicated by on average 10 dB higher average SMR thresholds compared with yNH listeners. That means, parsing musical scenes may be very difficult for oHI listeners, and future hearing devices may need to be optimized to account for this problem. The results may further suggest that in contrast to oHI listeners, yNH listeners were able to listen into the dips of the maskers. Listening in the dips could be a plausible strategy for yNH listeners to perceptually analyze densely packed polyphonic music, a process that warrants further research. We further observed that musical training was associated with an improvement of musical scene analysis abilities. However, there was no correlation between musical scene analysis abilities and SRTs, indicating that musical scene analysis entails auditory processing components that need to be studied in their own right. Given the restraints of oHI listeners’ musical scene perception, future work should more detailedly tease apart the individual effects of hearing impairment and age. Furthermore, paradigms such as the present one could be used as a starting point for comparing musical scene perception across various acoustic scenarios and hearing device settings. This may eventually provide a pathway into tailoring hearing devices for the intriguing complexity of real-world musical scenes.

## Figures and Tables

**Figure 7.**
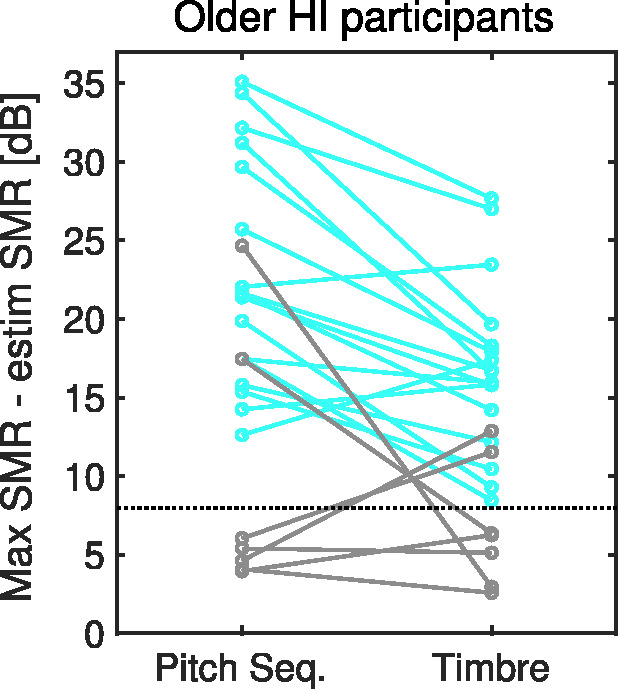
Maximal SMR minus estimated SMR thresholds for oHI participants in the pitch sequence and timbre tasks. The dotted line indicates the cutoff of 8 dB. Gray lines correspond to discarded participants.

## Appendix A: Musical Training Self-Report Inventory

The following self-report items (and corresponding weightings in brackets) were used to assess musical training (Müllensiefen et al., 2014): Number of instruments played (0.82), having been complimented on performances (0 = *never*, 1 = *always*; 0.72), number of hours practiced in period of peak interest (0.71), years of music theory training (1.43), years of instrument training (1.67), considers self-musician (0 = *fully disagree*, 1 = *fully agree*; 0.90).

## Appendix B: Presentation Level

We did not assume level to play a great role for yNH participants, which is why we fixed the masker level for yNH listeners at 65 dB SPL. On the contrary, for oHI participants, it seemed critical to ensure audibility and a comfortable listening level by individualizing masker levels. Using the BTUX fitting method (Oetting et al., 2014), the mean medium loudness estimates (25 CU) of yNH listeners were 70.8 dB SPL (*SD* = 7.3) and 76.2 dB SPL (*SD* = 8.0) for oHI listeners. That is, for yNH listeners, the presentation level of the masker at 65 dB SPL deviated by around 5 dB from the yNH group average level, whereas the masker levels for oHI listeners were matched per participant (rounded in 5 dB steps). It could thus be argued that advantages of yNH over oHI participants could be due to the comparatively lower presentation levels. This would be consistent with reports of increased pitch discrimination thresholds as a function of increasing presentation level (Bernstein & Oxenham, 2006). In the present study, however, we do not think that the masker level played a great role. To obtain an estimate of the effect of masker level, a control experiment using the pitch sequence task with the cello masker was run with four musically trained yNH participants. Covering the full range of levels from the main experiment, the masker was adjusted to 65, 72.5, or 80 dB SPL. As in the main experiment, thresholds were measured twice and presented in random order. Notably, we did not observe any trend based on masker level: Mean SMR thresholds (range = –25.8, –14.9) were very similar with –20.2 (*SD* = 1.5), –20.0 (*SD* = 3.9), and –19.9 (*SD* = 3.0) dB SMR for the three masker levels of 65, 72.5, and 80 dB SPL, respectively. In conclusion, effects related to masker level do not seem to be strong or consistent across yNH participants.

## Appendix C: Headroom

To estimate SMR thresholds with an adaptive procedure, there should be sufficient *headroom* for the staircase, that is, participants’ thresholds should be well below the maximal SMR for the adaptive procedure to be reliable. In the present experiment, the maximal presentation level of the signal was limited to 90 dB SPL, which implies that oHI participants with medium loudness estimates of 80 dB may encounter a maximum SMR of 10 dB, participants with loudness estimates of 70 dB may encounter maximal SMR values of 20 dB, and so forth.

Figure 7 shows the distribution of differences of maximum SMR values and estimated SMR thresholds for the pitch sequence and the timbre tasks. The figure indicates that there was indeed a bimodal distribution for the pitch sequence task, with five older HI participants exhibiting average headroom values (i.e., maximum SMR minus the estimated SMR threshold) of less than 8 dB (i.e., four times the final step size). For the timbre task, headroom values from five participants were below 8 dB. Overall, the data with headroom values below 8 dB in either the pitch sequence or the timbre task stemmed from seven participants that we decided to discard in the main analysis because it was questionable whether the corresponding estimated threshold values were accurate or meaningful.

In comparison with a model with the full set of participants, the statistical model for the reduced set of oHI participants yielded smaller effect estimates for the variables group and training with coefficients shrinking from β = 6.3 to β = 5.0 and β = –2.6 to β = –1.6, respectively, but there were no other notable differences.

## Appendix D: Model Statistics

In the following, key statistics are listed from the statistical analysis of the musical scene analysis tasks. The musical training factor was continuous and *z*-normalized; all other factors were dichotomous and sum-coded.

## Note

1
www.vsl.co.at
